# In silico toxicology: From structure–activity relationships towards deep learning and adverse outcome pathways

**DOI:** 10.1002/wcms.1475

**Published:** 2020-03-31

**Authors:** Jennifer Hemmerich, Gerhard F. Ecker

**Affiliations:** ^1^ Department of Pharmaceutical Chemistry University of Vienna Vienna Austria

**Keywords:** adverse outcome pathway, computational toxicology, in silico toxicology, machine learning, read across

## Abstract

In silico toxicology is an emerging field. It gains increasing importance as research is aiming to decrease the use of animal experiments as suggested in the 3R principles by Russell and Burch. In silico toxicology is a means to identify hazards of compounds before synthesis, and thus in very early stages of drug development. For chemical industries, as well as regulatory agencies it can aid in gap‐filling and guide risk minimization strategies. Techniques such as structural alerts, read‐across, quantitative structure–activity relationship, machine learning, and deep learning allow to use in silico toxicology in many cases, some even when data is scarce. Especially the concept of adverse outcome pathways puts all techniques into a broader context and can elucidate predictions by mechanistic insights.

This article is categorized under:

Structure and Mechanism > Computational Biochemistry and Biophysics
Data Science > Chemoinformatics

## Graphical abstract

How computers can help toxicologists' decision‐making.

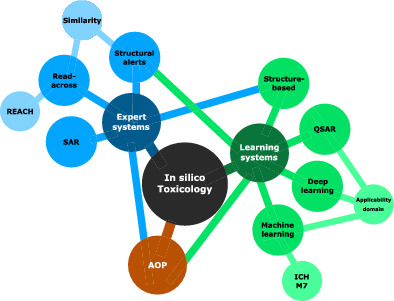

## 1 INTRODUCTION

Risk assessment is crucial and inevitable for pharmaceutical, cosmetics, and chemical industries as well as regulatory agencies. The goals of such assessments, however, are most diverse. In the pharmaceutical industries, risk assessment is conducted throughout the whole drug discovery and development process. Starting with the first evaluation of a compound, even before synthesis, it carries on until clinical trials where risk assessment is crucial for human health. Despite rigorous safety assessment of novel compounds, attrition still is high during the development process, often due to safety concerns.^1^ This is one of the reasons which prompted the European Federation of Pharmaceutical Industries and Associations (EFPIA; efpia.eu) and the European Commission to team up and found the Innovative Medicines Initiative (imi.europa.eu).^2^ This resulted in the launch of several large scale industry‐academia collaborative projects aiming at in silico prediction of toxicity, such as eTOX^3^ and eTRANSAFE (etransafe.eu).

In the chemical industries safety assessment is conducted as well, however, often with the primary goals being worker safety and environmental risk assessment. The cosmetics industry's risk assessments mostly revolve around consumer safety. Current safety testing in all industries is trying to implement the 3R principles which propose the reduction, refinement, and replacement of safety studies performed on animals.^4^, ^5^, ^6^ The announcement of EU regulation (EC) No 1907/2006 (widely known as REACH) and the European Union's Regulation (EC) No 1223/2009 (ban on animal testing in the cosmetics industry) shifted the focus from in vivo studies towards in vitro and in silico procedures for risk assessment. Another major step towards this goal was the European Pharmaceuticals Agency's (EPA) announcement of abolishing all funding for mammalian safety testing, as well as to eliminate mammalian safety testing itself from the approval processes by 2035.^7^

The aim of in silico toxicology is to predict certain hazards, such as mutagenicity or organ toxicities, based on computational models. These models can be created by experts (e.g., structural alerts or read across) or created automatically (e.g., machine learning techniques). The applicability of models ranges from the earliest stages of drug development—where a compound needs only to exist virtually to be testable—to risk assessment in time‐critical cases where in vitro or in vivo testing is not feasible, thus allowing for guidance on timely needed decisions. For example, the REACH legislation, where large amounts of chemicals needed to be tested for various apical toxicities, aimed towards in silico toxicology for gap filling techniques to deal with the impossible task of testing reams of chemicals within the given time frame. With in silico methods, hazards can be estimated early on, eliminating the need for synthesis. This can be especially important during drug development, where the in silico prediction of a compound can direct the choice for the lead compounds, not only concerning efficacy but also safety. Testing can, therefore, be directed towards specific hazards before a compound is taken forward to the preclinical safety testing. Furthermore, in silico toxicology could serve as a predictive tool for rare idiosyncratic events which can neither be seen in preclinical nor clinical safety assessments. For chemical safety assessment in silico models can be used to predict hazards for the users of such compounds, as well as for environmental safety.

The underlying assumption of all in silico toxicology approaches is the relationship between a chemical structure and its biological activity. This relationship states that the physiological action is a function of the chemical constitution, which was proposed by Brown and Fraser as early as 1868.^8^ A consequence of this is the “molecular similarity principle”,^9^ which states that similar structures will exhibit similar biological activities. This axiom was broadened over the last years by incorporating similarities based on gene expression or bioactivities.^10^, ^11^

While in silico toxicology has advanced significantly in the last years, in times of “big data” there remain plenty of opportunities for even greater leaps forward. Especially within regulatory frameworks its use still is quite limited and could be improved with the availability and FAIRification of new data.^12^ One example is the eTRANSAFE project, where preclinical toxicity data is integrated with clinical data (etransafe.eu). However, regulatory agencies currently only accept in silico predictions within the ICH M7 guideline^13^ for impurity testing and within gap filling for the REACH legislation. This is mainly due to the fact that until now not enough evidence and trust could be built up to reach the necessary confidence in such models.

In this review, we will give an overview of the different methods of in silico toxicology, especially with a focus on newly developed technology. We will discuss pitfalls and caveats for model building and frame our vision of the future of in silico toxicology.

## 2 TOXICITY TESTING

The goal of in silico toxicology is to help in risk identification, compound prioritization and ultimately, in combination with in vitro testing, to replace in vivo testing. Toxicological testing can be divided into general toxicity testing and mechanistic toxicity testing.^14^

### 2.1 General toxicology—saftey screening

Safety screening of novel drugs, chemicals, or other substances of concern (such as food contaminants) is generally conducted using in vitro and in vivo tests where a compound is administered in different doses and an effect has to be observed. For regulatory safety documents mostly in vivo tests need to be conducted. Such tests are conducted for two purposes: first, to determine the acute toxicity of a compound and second, to determine the repeated dose effects. Acute toxicity tests are usually used to determine the starting doses for organ toxicity testing.^15^ Repeated dose studies usually determine the toxicity of a compound. Mostly subacute repeated dose studies (28 days) and subchronic repeated dose studies (90 days) to determine organ (OECD TG 407 and 408),^16^, ^17^ inhalative (OECD TG 412 and 413)^18^, ^19^ or dermal toxicity (OECD TG 410 and 411)^20^, ^21^ are conducted in rodents. In some cases (e.g., for the REACH annex X^22^) long‐term toxicity studies (52 weeks) have to be conducted, however, this can be done in combination with a carcinogenicity study (OECD TG 452 and 453).^23^, ^24^

These tests are used to evaluate different exposure levels: The no observed adverse effect level which is a dose where the compound does not affect the organism and the lowest observed adverse effect level, which is the dose where first effects can be observed. These levels are then used to estimate either the first doses for clinical trials or the dose‐limits for human safety. However, such doses can only be estimated for noncarcinogenic and nongenotoxic substances. In addition to the required in vivo tests, there are many in vitro tests such as the Ames mutagenicity test or the skin sensitization assay, which can serve as a replacement for the in vivo tests. Further for specific chemical classes, such as agrochemicals, additional studies, like ecotoxicological assessment, have to be conducted. Additional information on regulatory toxicology can be found in References 25 and 26.

### 2.2 Mechanistic toxicology

Mechanistic toxicology revolves around investigating the reasons for substances to cause toxicity. This involves mechanistic in vitro as well as in vivo studies. Elucidation of mechanistic information involves illumination of the target as well as the pathways which lead to the apical toxicity endpoint. This knowledge can be used to gain deeper insights into the mechanisms of toxic substances. Furthermore, such knowledge can be utilized to prevent toxicity by avoiding specific structural features. Following mechanistic studies, cellular receptors and transporters, which can lead to certain types of toxicities, were identified. Such targets are called off‐targets, two of the most prominent examples are the human Ether‐à‐go‐go Related Gene (hERG) channel and the bile salt export pump (BSEP) transporter. Substances binding to hERG can lead to severe torsades des pointes^27^ and BSEP blockers often lead to cholestasis.^28^ Alongside (off)‐targets, (toxico)kinetics, especially in junction with genetic components, also plays an important role in the elucidation of toxicity mechanisms.^29^ Using kinetics, the exposure defines whether a hazard becomes an imminent risk. Similarly, the metabolism, and genetic variations thereof, such as fast and rapid metabolizers, can determine whether a patient develops severe side effects. In addition, genetic variants of the immune system might be responsible for severe idiosyncratic drug reactions.^30^ In the future, toxicogenomics might be able to help clarify such mechanisms further and develop a better understanding of pathways related to different apical toxicity endpoints.^31^ More detailed information on mechanistic toxicology can be found in Reference 32.

## 3 EXPERT METHODS

Expert methods use the knowledge and experience of experts to deduce or explain toxicity mechanisms. This can be done for single compounds as well as whole compound classes. Whereas read‐across is used to infer toxicity from other related compounds, structural alerts are a means to highlight potential hazards and help understanding the underlying mechanism. Expert methods are aimed at helping to assess compounds where no previous knowledge is available, therefore they can use mechanistic evidence as well as data from safety screenings to provide an assessment.

### 3.1 Read‐across

Read‐across is a method aiming at filling data gaps for a novel or previously uncharacterized compound.^33^ It relies on the formation of chemical categories from structures. The underlying assumption is that similar structures will have similar bioactivities.^9^ Therefore, read‐across is conducted by identifying similar molecules and evaluating their bioactivity, thus inferring the (non) toxicity of the parent compound. In the best case, the occurrence of a common scaffold makes it possible to analyze the influence of different substituents on the activity, allowing for a thorough assessment. Chemical similarity can be defined by calculating the similarity of feature vectors such as chemical properties or fingerprints using similarity metrics such as the Euclidean or Tanimoto similarity, opening up numerous possibilities for the calculation.^34^

Another part of the read‐across approach is the interpretation of available data, which is highly subjective and therefore there is no impartial solution. Due to the problem of chemical similarity and the subjective interpretation of the available data, read‐across was being regarded as an “ugly duckling” for methods that “originate from the idea that any information is better than no information in situations where there is no budget”.^35^ However, available data, as well as the use of the method is increasing. Especially the REACH initiative, which required large amounts of testing to be conducted, allowed read‐across for gap filling. The good news is that with “big data” a manifold of data sources is available to guide read‐across. Recently, Pawar et al. conducted a meta‐review identifying more than 900 databases that contain data relevant for read‐across.^36^

Several recent case studies demonstrated that a careful read‐across can be utilized to estimate the toxicities of various compound classes.^37^, ^38^, ^39^, ^40^, ^41^ However, all case‐studies show that the approach is highly dependent on the available data and the definition of similarity between the parent compound and the analogs. This is summarized in a final review of the conducted case studies by Schultz and Cronin who identified primarily transparency and uncertainties as being the main factor for a successful read‐across.^42^ In particular, they note “Today ‘chemical similarity’ means more than proving similarity in chemistry; it requires the category formation and RA [read‐across] process to be transparent, reproducible and clearly documented. Specifically, key principles of biological, as well as chemical, similarity need to be supported, where possible, by data and scientific evidence.”^42^ which emphasizes the high complexity of read‐across. To create a framework and guidelines for read‐across, the Organisation for Economic Co‐operation and Development (OECD), European Chemicals Agency (ECHA), and the European Centre for Ecotoxicology and Toxicology of Chemicals put efforts into guidelines for read‐across assessment.^43^, ^44^, ^45^ Especially many efforts were taken towards a reproducible and objective read‐across using mathematical models and quantifying the uncertainty of the prediction. The overarching conduction of read‐across consists of seven steps (based on Reference 46):

1. Decision context: what is the question to be answered.
2. Data gap analysis: where are knowledge gaps.
3. Overarching similarity rationale: wow is similarity defined.
4. Analog identification: based on the definition, which compounds are similar.
5. Analog evaluation: which similar compounds can be used.
6. Data gap filling: combine the knowledge gained from the analogs.
7. Uncertainty assessment: how clear is the evidence, where are still gaps, is the extrapolation adequate.

Thus far, many tools have been developed for category formation or read across (see also Reference 47). The ECHA made an effort to create a tool to guide, as well as asses, read‐across approaches and identify gaps and uncertainties. This framework is called the read‐across assessment framework (RAAF).^48^ It establishes different scenarios for the assessment of similarity and the subsequent deduction of evidence. This is important because especially compound similarities, evidence, used data and lack of plausibility were identified by Ball and coworkers as main reasons for rejected read‐across studies.^49^

Recently, the focus was shifted from purely chemical similarity towards using auxiliary bioactivity fingerprints for a better characterization of the underlying mechanisms.^10^, ^50^, ^51^, ^52^, ^53^ One approach, called GenRA, uses a bioactivity based similarity derived from different toxicity endpoints. Helman and coworkers could show that GenRA achieves a better predictivity when applied to structurally‐related clusters of chemicals.^54^ Recently, GenRA was also implemented in the EPAs ToxDashboard.^55^

The most important step of a read‐across, besides the assessment of the compound similarity, is the assessment of the remaining uncertainty. For uncertainty assessment, many analyses were done defining different sources such as the similarity of the analogs, confidence in the underlying data or the weight of the available evidence. Schultz and coworkers made a great effort to characterize those various sources.^56^ Their analysis resulted in 30 questions that target all the uncertainties and thus allow a very thorough estimate. In addition, they evaluated existing read‐across frameworks and conclude that currently, the RAAF incorporates most of these questions. New approaches for read‐across include the use of gene expression data^57^ or the implementation of local quantitative structure–activity relationships (QSARs) for the prediction of the compounds.^58^ However, especially the QSARs suffer from having a low explainability and thus contradict the concept of the transparency of a read‐across. Nevertheless, local QSARs, especially with explainable algorithms, can help in assessing the toxicity in addition to the read‐across if sufficient data is available. For more information see Reference 59.

### 3.2 Structural alerts

Structural alerts, like read‐across, are based on the idea that chemicals can be grouped into clusters of molecules exhibiting similar toxicity and, moreover, having a similar mode of action. A structural alert is a common substructure that can be linked to a certain type of toxicity. Such alerts can be derived through expert knowledge^60^ or by statistical evaluation of fragmented datasets (e.g., References 61, 62, 63, 64, for a comparison see Reference 65). The statistical evaluation of a dataset is usually done by utilizing a fragmentation algorithm and subsequently deriving structures that are associated with certain toxicities. For this, the user assumes that, if a substructure is present in a higher percentage of toxic than nontoxic molecules, the structure might be responsible for the toxicity. Floris and coworkers complemented this approach by laying out a basis for the statistical analysis of newly found alerts, which is using the chi‐squared test on an alert's contingency table.^66^

However, often no mode of action can be directly assigned to such statistically found alerts. Derivation of alerts by expert knowledge yields the advantage of an explanation for an alert. This explanation often results in mechanistic insights that subsequently can be used to suggest structural changes, where applicable.^67^ Independent of their source, structural alerts can help in the identification of hazards. Alerts can cluster datasets according to a potential common mechanism of such compounds.^68^ In addition, they yield a starting point for mechanistic studies^69^ or structural changes^67^ to minimize the risk of toxicity. However, as alerts only see one part of a structure, they fail at analyzing the different effects of multiple groups occurring at the same molecules. Those effects might be influencing each other and thus influence the toxicity. This is, for example, known for the induction of mutagenicity of nitro groups. Although widely known as a structural alert, the nitro group is often used in therapeutics (e.g., cancer therapeutics) as a functional group providing reactivity towards the target.^70^ However, it is also known that by structural modification of such compounds the genotoxicity can be reduced, if not abolished.^71^, ^72^

A new method, which is able to partly incorporate such modifications is the generation of structural alerts through scaffold trees.^73^ The generated trees show how the hazard of a molecule changes when the parent fragment is modified. When assessing a compound with structural alerts it is important to understand the absence of an alert does not denote a molecule to be nontoxic, rather it states no known alert could be found. However, with sufficient available data this can be made possible.^74^ It was also shown that the potency of an alert might be corresponding to the daily dose, therefore alerts might not be relevant for low doses of drugs.^75^ Alves and coworkers therefore proposed that structural alerts should not be seen as a ”yes” or ”no” concerning toxicity but rather as a hypothesis about the mode of action and subsequently trigger closer mechanistic studies.^67^, ^76^ Similarly, Limban and coworkers propose the replacement of structural alerts by functional groups that counteract the supposed toxicity mechanism rather than directly rejecting such compounds.^67^ Kalgutkar also highlighted, that, although structural alerts should not be ignored, especially in the case of toxicity via metabolic activation, alerts should be used with care^77^ as metabolic activation might be heavily influenced by existing residues of a scaffold. Myden and coworkers also showed that structural alerts can be highly sensitive, leading to many false‐positive results.^78^ Overall, structural alerts serve as a means to group chemical structures and to derive common mode of actions. However, as alerts do not consider the whole molecule and do not allow for truly negative predictions, they should only be used to indicate a hazard which can be used to guide future studies.

## 4 MACHINE LEARNING BASED PREDICTIONS

### 4.1 2D and 3D quantitative structure–activity relationships

Quantitative structure–activity relationship (QSAR) analysis is the advancement of SAR analysis. SAR analysis dates back to a thesis of Cros who discovered that toxicity increases with diminishing water solubility.^79^, ^80^ By SAR analysis of congeneric series, it can be determined how substituents influence the bioactivity of a compound. In 1963 Hansch and Fujita published a first mathematical method termed *p* − *σ* − *π* analysis to correlate changes in biological activity with changes of structural properties.^81^ This was complemented by Free and Wilson's mathematical contribution to structure–activity relationships.^82^ With both methods, it became possible to have a quantitative hypothesis for the contribution of different substituents to the biological activity. However, it needs to be noted that in traditional QSAR only distinct congeneric series are analyzed. This is mainly because QSAR was intended to provide information on the physicochemical features required at specific positions of a chemical scaffold in order to enhance binding affinity. Basic requirements for a traditional QSAR analysis thus require a concrete ligand–receptor interaction, presumably in the same binding mode. Only then changes in for example, electronic properties of an R group can be related to changes in the biological activity of the compound.

In the early times of QSAR, descriptors and fingerprints derived from the 2D structure of the compounds were used as an input feature vector. With the increasing computational power available, also the use of methods based on three‐dimensional representations of molecules became possible. Nevertheless, this comes with the cost of intense conformational sampling requirements. In alignment‐dependent 3D‐QSAR methods such as CoMFA^83^ and CoMSIA,^84^ each compounds' conformational space needs to be sampled to derive the energetically most favorable conformation, and these need to be aligned. In alignment independent methods, such as GrIND,^85^ VolSurf,^86^ Pentacle,^87^ and 3D autocorrelation vectors,^88^ descriptors are calculated from distinct 3D conformations of the individual molecules and used for correlation analysis. Although in this case no alignment is necessary, the methods still require a thorough conformational analysis, as the descriptor values are conformation‐dependent.

Considering all this, it becomes evident that classical 2D‐QSAR and 3D‐QSAR are not considered to be a valuable method for predicting toxicity, especially in case of complex in vivo endpoints such as hepatotoxicity, cardiotoxicity, or neurotoxicity. In these cases, multiple physicochemical (solubility, crossing barriers) and molecular (interaction with a transporter, cytochromes, off‐targets) events are involved, which preclude any traditional QSAR method other than correlation with very global physicochemical features such as logP, TPSA, or number of H‐bond donors, ‐acceptors, or rotatable bonds. However, for very specific endpoints, and if congeneric series are available, QSAR and 3D QSAR can be used to determine properties decreasing the toxicity.^89^, ^90^, ^91^, ^92^

### 4.2 Structure‐based approaches

With the increasing amount of protein structures deposited in the Protein Data Bank (pdb; http://www.rcsb.org/),^93^ also structure‐based approaches are used to predict toxicity. However, in this case there needs to be a clear, causal link between a protein (in this case called off‐target) and the adverse effect. One of the prototype examples in this respect is the hERG potassium channel and its link to the long QT syndrome. Although recent work demonstrates that blocking hERG not necessarily leads to Torsades de pointes,^94^ affinity to hERG is routinely tested in the drug discovery and development process. This need also triggered the development of respective in silico methods. Besides numerous ligand‐based models applying basically all methods used in machine learning,^95^, ^96^, ^97^, ^98^, ^99^ also structure‐based approaches such as docking into protein homology models of hERG were explored. These studies comprise, for example, induced fit docking of a small set of hERG blockers,^100^ insights into the molecular basis of drug trapping of propafenones in hERG,^101^ up to calculation of absolute binding free energies between hERG and structurally diverse ligands.^102^

Also, for other off‐targets such as ABC‐transporter structure‐based methods were applied to get a deeper understanding of the molecular basis of compound/off‐target interaction. In case of P‐glycoprotein, SAR guided docking^103^ led to a docking protocol which allowed to perform structure‐based classification of a large set of P‐gp inhibitors and noninhibitors.^104^ Also for the BSEP, an ABC‐transporter linked to cholestasis, structure‐based classification was successfully implemented.^105^ Finally, we would like to mention the off‐target safety assessment (OTSA) framework, which combines a number of 2D target prediction methods with 3D protein‐based approaches. Briefly, the combination of six different cheminformatic methods with 3D‐methods such as pocket descriptors allows to predict off‐target interactions for small molecules.^106^ While OTSA basically predicts potential interactions, proteochemometric modeling tries to predict quantitative compound‐target interaction values by including target‐based descriptors into the data matrix for classical QSAR analyses.^107^

### 4.3 Traditional machine learning

Over the years the original QSAR analysis was expanded by different groups. Today, in the field of in silico toxicology, the term QSAR is used very widely to describe all forms of predictive modeling, including more complex (and less quantitative) machine learning models. Machine learning is a term comprising supervised model building for classification and regression, unsupervised techniques such as clustering, and reinforcement learning techniques mostly applied to sequential decision making tasks such as molecular design.^108^ In the following, we will use the term machine learning to refer to supervised learning for classification or regression models if not stated otherwise. Machine learning is split into two ”worlds.” Traditional machine learning refers to techniques such as k‐nearest neighbors (kNNs), random forests, or support vector machines, which are separated from neural networks as the central algorithm of deep learning (which will be discussed in the next section).

To train traditional machine learning models, datasets of 100 compounds or more should be used. For training, the molecules have to be transformed into a suitable representation such as descriptors or fingerprints. Descriptors usually constitute molecular properties such as the log *P* or the number of hydrogen bond donors, but can also involve more complex 3‐dimensional properties such as atom distances or surfaces. Fingerprints are bit‐vectors or count vectors which report the presence or absence or the number of occurrences of a structural feature. These representations are then used as input for model generation. By trying to predict the training set molecules correctly, and reporting the errors made back to the model, the model is improving its prediction.

A model can either be a regression model, predicting a continuous variable such as the LD50, or a classification model, such as a model for mutagenicity. Classification models are usually predicting a binary outcome or a continuous value between 0 and 1. Such continuous values can either be transformed into a binary outcome using a threshold (commonly 0.5) or can be transformed into a probability. Lately, many developments focus on thorough validation procedures to increase regulatory acceptance and to build trust. In this respect the main concerns are related to the limited chemical information used for building the model, which reduces the confidence in prediction of dissimilar molecules. Frenzel and coworkers showed that freely available Ames models can especially help in the prioritization for mutagenic or carcinogenic heat‐induced food contaminants. However, they concluded that the evaluated models are not yet predictive enough to use them as a stand‐alone tool.^109^ In contrast, a large international study revealed that existing and commonly used models for Ames mutagenicity are working well and the predictivity can compete with the in vitro Ames test.^110^ In this context it has to be noted that, depending on the dataset used, it might be that the contaminants Frenzel and coworkers used are too specialized to be caught by models in the public domain, which usually also only rely on public data. Thus, their molecules might not be in the applicability domain (see also Section 4.5). Another important finding by Honma and coworkers was that incorporating newly available data also increases the performance and thus models should be updated regularly.^110^

Another important step is leveraging available (big) data to build better machine learning models. This was shown by Luechtefeld et al. who mined available data from regulatory agencies and developed RASAR, reporting to outperform animal test accuracy.^111^ Although the methodology was criticized later,^112^ this approach highlights the need to leverage the available data and combine available datasets. Besides that, data mining, especially from nontabular data such as study reports, can increase the availability of data and thus improve existing models.

Unfortunately, developed models often lack an explanation of the prediction. Yet, this is one of the important prerequisites in the OECD's QSAR validation guidelines (see Section 4.6). The last point states that models should have a (mechanistic) explanation to be widely accepted. Usually, models are black boxes and, apart from the input and the output, the user does not know what is happening internally. Some models (such as regression or random forests) can at least explain the most relevant input features. Therefore, the focus has shifted from the mere model building towards establishing trust in a model's prediction, by giving confidence estimates for a prediction. In particular, conformal predictions can yield such an estimate of the certainty of the model for a specific prediction.^113^, ^114^ In brief, a conformal predictor is built using an existing model along with a calibration dataset. Predictions of this dataset are then used to determine the *p*‐value of a new prediction. This *p*‐value is calculated for both classes (i.e., toxic or nontoxic) separately. If the *p*‐value is above the chosen significance level the compound belongs the class. Possible outcomes of conformal predictions are a compound belonging to (a) only one class, meaning the compound is classified with high confidence, (b) both classes, denoting the compound cannot be predicted with high confidence, or (c) it belongs to no class, denoting that the compound is too dissimilar and cannot be predicted.

The last option, therefore, represents a way to get confidence scores and using them as an applicability domain for the models.^115^ In addition, it was shown that conformal predictions can also be used to deal with imbalanced data, which is a common problem for toxicological datasets.^114^ Another method to convert predictions into real probabilities is Platt scaling, which uses a learned log transformation to calculate the class probabilities from existing predictions.^116^ A very interesting approach to interpretability was introduced by Alves and coworkers. Their approach, Multi‐Descriptor Read Across, uses a kNN approach to classify compounds. The novelty lies in using four different types of descriptors to determine the nearest neighbors.^117^ Using the kNN method a prediction can be intuitively understood by inspecting the nearest neighbors used by the algorithm. The drawback of all traditional machine learning methods is that missing data has to be imputed to be able to use incomplete data matrices.

### 4.4 Neural networks and deep learning

Neural networks are a technique dating back to the 1940s and 1950s.^118^, ^119^ They have long been used to predict different tasks. However, it was not until 2006 that their real breakthrough started.^120^, ^121^, ^122^ With the availability of graphical processing units, training of neural networks has been hugely facilitated yielding new opportunities.^120^ Since neural networks now contain much higher numbers of hidden layers the technique was re‐branded as deep learning. For bioactivity predictions, the Merck Kaggle challenge and the Tox21 challenge showed that neural networks might be superior to traditional machine learning, yielding higher predictive performance and thus winning the challenge.^116^, ^123^, ^124^, ^125^

**Figure 1:**
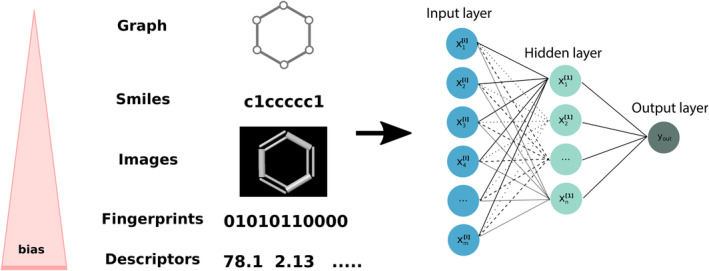
Scheme of a neural network with possible inputs. The order of the input from up to down, is in concordance with the bias introduced by the user

The biggest advantages of deep learning for bioactivity/toxicity predictions are (a) the flexibility with regard to the structural representation and (b) the possibility of multi‐task predictions (i.e., predicting multiple toxicities in the same model). In fact, deep learning is always seen as a method able to extract meaningful features by itself, thus not needing any feature generation beforehand. This can be impressively seen in the image recognition field.^126^ For the prediction of chemicals, this implies, instead of using descriptors, the molecules themselves should be used as an input. Effectively, this opens doors for predictive toxicology (see Figure 1). By providing images, molecular graphs, 3D grids, or SMILES strings, the network can learn the necessary properties or patterns by itself, based on the assumption that all information needed is encoded in the structure.^127^ Especially in the case of graphs, smiles or 3D grids, the model is not biased by the user's selection of descriptors, which might not be suitable for the task at hand. Such approaches have yet to be extensively studied.

Nevertheless, a few pioneers already obtained interesting results. Goh et al. developed Chemception, a network predicting toxicity from 2D images of chemical structures, and later asked: “How much chemistry does a network need to know?”.^128^, ^129^ The results showed that training can be conducted with images but encoding additional chemical knowledge is helpful for the predictive performance. Similarly Fernandez et al. trained a network solely on images and reported comparable performances to other state‐of‐the‐art models.^130^ Although training on structural images might not be the goal for predictive toxicity, image training could purposefully be used in the detection of toxicities from pathological or image‐based techniques. Jimenez‐Carretero et al. showed that convolutional neural networks can be used to predict toxicity from cell staining images and even classify them by their mode of action.^131^ Another study was able to predict assay outcomes from microscopy images from high throughput screening studies.^132^

Apart from images, SMILES can also be used to predict the toxicity of a molecule, as implemented in SmilesNet developed by Gini and coworkers. It uses SMILES strings as an input, transforms them into a feature vector, and subsequently predicts the mutagenicity of a compound.^133^ A representation which seems to be the most natural in light of representing molecules as atoms and bonds is the molecule representation by graphs. In such graphs, the atoms are represented by the vertices and the bonds are represented by the edges between them. Xu et al. showed that the prediction of acute oral toxicity can benefit from a graph representation.^134^

Two very interesting approaches which do not use chemical structures as an input, but interpret in vitro assay outcomes are the prediction of seizure induction by a compound^135^ and the prediction of liver toxicity endpoints by outcomes from transcriptomic data.^136^

Another discovery which has highly impacted bioactivity prediction is the use of multitask networks.^137^, ^138^, ^139^ These networks predict multiple toxicities at once. It was shown that they benefit from the multitask setting by increasing the performance and at the same time regularizing the network to prevent overfitting. Tasks used in such approaches do not have to be closely related, however, a commonality of such tasks, like for example the same mode of action, should be suspected to benefit from multitasking.^140^ Without any commonality or similar structures, the model cannot make use of a shared representation for the tasks which is without benefit for training.

This is of great interest to the toxicological community since it is known that compounds can have multiple targets and mode of actions and thus might not be constricted to causing one single toxicity. Moreover, the method does not need to be used on a completely filled outcome matrix, but compounds can have missing activities for some endpoints and nonetheless be used for the predictions. This yields much larger datasets which can be used for better training of models. Many studies already highlighted that multitask networks exhibit better predictivity than single task networks. The application so far was done for modeling of acute toxicity,^134^, ^141^ reactivity,^142^ and ADME‐Tox properties.^143^ All this work highlights, that without the need to use descriptors or fingerprints to transform a molecule into a suitable input of machine learning models, in silico toxicology can benefit greatly. The possibility to combine related endpoints to form larger datasets is also a very important step towards larger, and hopefully more generalized, datasets.

Although all methods are very promising, the problem of the interpretability still exists, especially for multitask networks. However, interesting conclusions were drawn by Mayr et al., Gini et al., and Xu and coworkers who could show that networks can learn representations which are comparable to structural alerts.^116^, ^133^, ^134^ Wenzel and coworkers introduced response maps to highlight important features used by the network.^143^

### 4.5 Applicability domain

Models are trained on a dataset representing a certain part of the chemical space. The aim is to generalize a model such that it can possibly predict all available chemical compounds. However, looking at the currently known organic chemical space which was estimated at 3.4 × 10^9^ 144 or even more compounds and the dataset size, which usually lies between a few hundred to a few thousand molecules, it becomes clear that even training with a large dataset of 10,000 compounds means that only 0.00003% of the chemical space is covered. Therefore, a model can never reliably predict the whole chemical space and predictions should be confined to the chemical space used for model building. The concept of comparing newly predicted molecules to the training set, as well as giving confidence about the new prediction, is called the applicability domain. This important criterion is also included in the OECD guideline for the validation of QSAR models (see Section 4.6).

In their very comprehensive review of applicability domain methods, Mathea and coworkers divided the concept of applicability domain into novelty detection and confidence estimation.^145^ They define novelty detection as checking whether an unseen compound is part of the chemical space the model was trained on. Confidence estimation is defined as the reliability of a prediction, therefore denoting how confident a model is that the predicted class is correct. The separation into the descriptor and predictive domain make an interpretation of a prediction for an unseen compound comprehensible and traceable.

**Figure 2:**
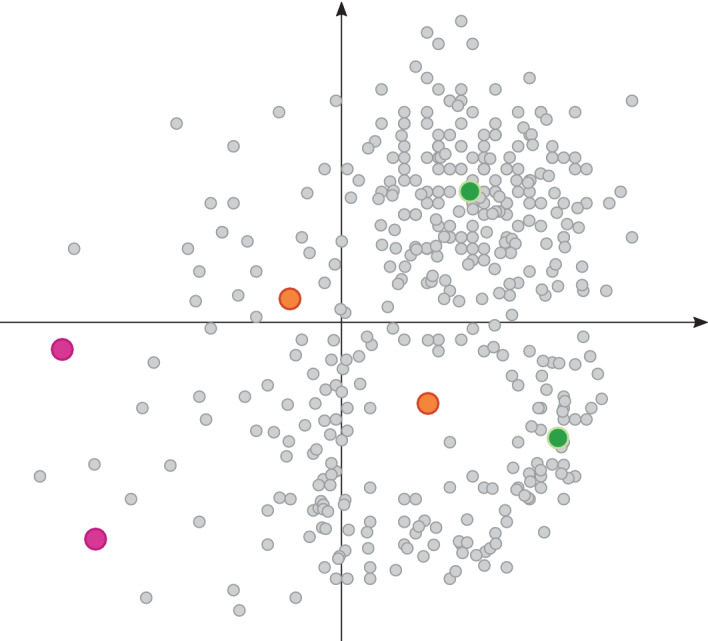
The chemical space of a QSAR model. The grey dots represent compounds from a hypothetical training dataset. The colored dots represent compounds which should be predicted by the model. The green dots represent compounds that are within the applicability domain. The pink dots represent compounds that would be predicted as out of domain as they lie on the borders of the chemical space which is very little populated. For the orange compounds, it is not clear from visual inspection how confident the model is in the extrapolation of such compounds as the surrounding area is populated, the compound, however, lies in a gap in the models' chemical space. Different applicability domain calculation might give different results here

Hanser and coworkers expand this concept into three stages: they term the novelty detection as applicability and split the concept of confidence estimation into a reliability and decidability estimation.^146^ Reliability estimation uses the compound neighborhood to determine if there are data points close enough and how well the model predicts these data points. The decidability is the weight of evidence that the prediction of the novel compound is correct. This could, for example, be a posterior probability or the evidence from single decision trees in a random forest. Both publications highlight the importance of dividing the applicability domain into two areas, namely the descriptor space and the predictive space. Both add valuable information about the prediction and can inform the user how trustworthy a prediction is. Thus, aligning with the aforementioned methodology, the modeler should give users an estimate of how close the predicted molecule is to the training dataset. Only if the predicted molecule shares a certain similarity, a model can reliably extrapolate from the seen values (see Figure 2). For example, a model trained on a quinone dataset will be able to predict quinone‐like molecules, however, it might fail to predict benzopyrene due to the limited structural similarity.

This again leads to the question of the definition of structural similarity. As already noted, assessing structural similarity is highly complex and always dependent on the molecular representation. For small datasets, a trained medicinal chemist might be able to define the represented chemical space by visual inspection, whereas for large datasets and newly predicted molecules this needs to be automated. Independent of the method, the chemical similarity of a compound to the training dataset should always be measured based on the model input such as fingerprints or descriptors. As secondary information, the modeler should give the user as much information as possible on the closest compounds in the training set^146^ as well as a confidence score, which is inherent in most used classifiers.^145^

Roy and coworkers recently published an applicability domain calculation that implements all three criteria proposed by Hanser and coworkers. They use the prediction error of the 10 nearest neighbors in cross‐validation, the similarity to the training dataset, and the proximity of the predicted activity to the mean of the training data.^147^ Although they finally develop a combined score, their findings are in line with the conclusion from Hanser and coworkers, that a combination of applicability, reliability, and decidability is beneficial as all contribute to a successful prediction. Conformal predictors as introduced before are using their means of generating out of domain prediction. They can fail to confidently assign a class to a prediction, therefore, the user knows the compound is out of domain.^115^ This estimate can be considered as information about reliability.

In addition to the question of how an applicability domain can be successfully implemented, it is also interesting to see which models need a defined applicability domain. Liu and coworkers showed that, while it is generally accepted that traditional machine learning models need an applicability domain, deep learning models also cannot generalize much better.^148^ However, this is not very surprising given that until now most deep learning models are not built on much larger datasets than traditional models. In the future, with the availability of bigger datasets, this might change in favor of a more relaxed definition of the applicability domain.

### 4.6 Regulatory frameworks for QSAR

In general, QSAR is not only an old but still evolving field as can be seen by the more extensive use and thus the efforts to harmonize and increase the use. In 2007 the OECD published guidelines on the validation of QSAR models.^149^ The guidelines demand models which conform with the following principles:

1. a defined endpoint,
2. an unambiguous algorithm,
3. a defined domain of applicability,
4. appropriate measures of goodness‐of‐fit, robustness, and predictivity,
5. mechanistic interpretation, if possible.

These guidelines also highlight one of the biggest pitfalls of QSAR modeling: While it is easy to obtain a predictive model, it is much harder to ensure that the predictive model is useful. Whereas the first two principles—the endpoint and the algorithm—can be managed with little effort, the last three points need thorough consideration. The applicability domain, as discussed earlier, is important since the chemical space is too large for a model to be wholly generalized. Thus, a measure is needed to evaluate the similarity of a compound to the training set as well as a possibility to obtain a reliability for the prediction. Point number four—the performance—is probably the most important point. Model evaluation should not only be conducted thoroughly, that is using an external test set to obtain the performance on a nonrelated structural dataset, but also should use appropriate measures. The last point—a mechanistic explanation—is not obligatory for a model. However, in addition to the applicability domain, mechanistic information can not only inform a user but also help to evaluate a developed model further.

With these principles available for model development, in 2014 the ICH M7 guideline was published. The revised ICH M7 guideline for the mutagenicity assessment was a big step towards the use of in silico approaches for regulatory documents. This guideline was a milestone for in silico toxicology as it represents the first guideline to allow the replacement of an in vitro test with an in silico prediction. Furthermore, this guideline is accepted by many regulatory agencies.^150^ It allows the assessment of manufacturing impurities of low quantity employing two orthogonal approaches. These approaches usually are one expert tool, relying on structural alerts, and one machine learning model. Both models have to be complementary and have to comply with the OECD validation principles.^151^ If both systems are predicting the compound as negative, no in vitro Ames test has to be conducted. For an ambiguous outcome—for example, contradicting predictions, an out of domain or inconclusive prediction—the expert review plays a crucial role for further decisions.^152^, ^153^ The expert review can, by adding additional evidence, clarify the prediction and make a final decision. In addition, it can also be used to refute a positive prediction, but substantial evidence is needed.^152^ This application of in silico prediction also highlights the need for certain negative predictions and the applicability domain. For instance, the absence of a structural alert does not necessarily denote that a compound is inactive. In combination with the applicability or novelty detection, for example, by looking at the closest negative compounds, this negative prediction can, however, be justified as a true negative.^74^

Apart from being a milestone, the ICH M7 guideline has also influenced other guidelines such as the toxic substances control act^154^ or the International Standard ISO 10993‐1 for the biological evaluation of medical devices, which both implement the possibility for in silico assessments.

## 5 ADVERSE OUTCOME PATHWAYS

**Figure 3:**
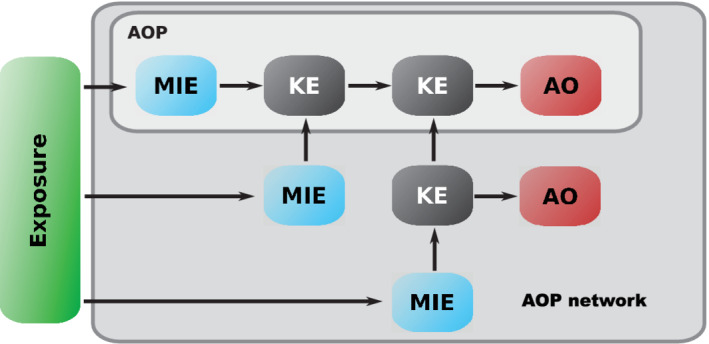
Schematic drawing of an adverse outcome pathway. The pathway consists of a molecular initiating event (MIE), several key events (KE), and an adverse outcome (AO). The light grey area depicts a scheme for a linear, single adverse outcome pathway. The dark grey area depicts an adverse outcome pathways network with multiple initiating events and multiple adverse outcomes. The green square with exposure highlights that this information is not yet part of the AOP framework, however, it is highly interlinked

The goal in toxicology has shifted from a mere determination of the hazard of a compound towards a mechanistic explanation. This explanation enables structural changes as well as an extrapolation of the risk of a compound (e.g., is the mechanism relevant only if a compound was ingested or can skin contact lead to sensitization, etc.) and thus a better safety profile of drugs as well as chemicals. Thus far we have explained how different in silico techniques can be used to determine hazards emanating from a compound. In principle, two events usually are described by such models: first, the direct interaction of the compound with cellular organelles or compartments—like for example the binding of a compound to DNA—or second, the resulting apical endpoint, such as carcinogenicity, which is commonly used for hazard evaluation. The first concept is also termed molecular initiating event (MIE), and the second is termed adverse outcome. The event cascade which is following the MIE is referred to as an adverse outcome pathway (AOP), the events are referred to as key events (see Figure 3).

These pathways were first introduced for ecotoxicology.^155^ ^1^ The AOP framework allows the assessment of chemical hazards through the incorporation of mechanistic information. This information can elucidate species differences or account for causal evidence of found statistical correlations. Very useful resources to discover known AOPs are the AOP Wiki (aopwiki.org) and the AOP‐KB (aopkb.org) or the OECD series on AOPs.^156^

An important property of AOPs is their independence from a specific substance. Therefore, an AOP can be used for all compounds where mechanistic evidence is available. AOPs can either be generated manually by literature review or mechanistic studies or by automated data mining.^157^, ^158^, ^159^ As mentioned earlier, high throughput screening data is a valuable tool to advance in silico toxicology. Fay and coworkers showed that high throughput screening data can also be used to identify MIE or key events for AOPs and help prioritization of testing resources.^160^ Incorporating mechanistic information can highlight data gaps or pave the way towards specific testing. Such tests can then be utilized in early phases of drug development to test a compound for the activation of AOPs, which might be a crucial factor in the decision process for the advancement of a novel compound.

To a certain extent, this is already done by specific testing for off‐target activities such as hERG‐channel blocking, which is known to increase the risk of QT‐elongation in drug‐users. With the availability of more MIEs, this safety panel could largely expand and focused in vitro testing approaches could be developed. Furthermore, general toxicogenomics data is already used (although infrequently) in mechanistic toxicology studies to elucidate the mode of action.^161^, ^162^ Thus it could be used to elucidate the mechanisms and help the development of AOPs, as well as, AOP networks.^163^ AbdulHameed and coworkers mined several toxicogenomics datasets and integrated the information in their assessment of the AOP for cholestasis and thereby identified mitochondrial toxicity as a, so far, overlooked cause of cholestasis.^164^

Yet, information of an AOP is limited to a potential hazard. For risk assessment, the AOP framework can only be used when linked with toxicokinetic data^165^ or as suggested by Escher and coworkers linking the AOP to the exposome and aggregate exposure pathways (AEPs)^166^ (see Figure 3). In a case study, Hines and coworkers showed that AEPs indeed can be beneficial to human risk assessment.^167^ Quantitative AOPs were proposed by Conolly and coworkers and extend the concept of AEPs by including dose‐ and time‐response information.^168^

Apart from being a self‐contained method, AOPs can also be linked to the above mentioned in silico methods. Structural alerts can, first of all, categorize compounds and subsequently link those groups to specific MIEs if the alerts mode of action is known.^68^, ^169^ In a read‐across analysis, AOPs can be incorporated as an additional layer of information. In the assessment of compound toxicity via traditional machine learning or deep learning, the AOP concept can also be very useful. Machine learning can be used to predict the MIE or the AOP of a compound, and in principle all intermediate steps as well, to guide the safety assessment.^170^ However, it has to be noted that the MIE, as well as the adverse outcome, have large differences in the model quality. The MIE is a very specific and well‐defined event, where usually structural determinants are known or can be deduced by knowledge of the target structure. The adverse outcomes are often much harder to model. One reason is the lacking clarity of the endpoints, as data is often based on the interpretation of an outcome. For example, in the case of drug‐induced liver injury, the data is based on case reports, which, for example, might be biased by the examiner or via unknown multidrug use. In animals, the outcome is based on the histopathologist's interpretation of the seen lesion which might vary between individual assessments. The second reason is that one adverse outcome, such as drug‐induced liver injury, can be caused by a multitude of MIEs. Thus, the model needs to be able to interlink all possible causes with the outcome, which is more complex as the structural variability is much higher.

Nevertheless, both events can be modeled, however, with different purposes. Whereas the MIE can guide early hazard assessment towards specific assays or tests, the adverse outcome can be used, in combination with toxicokinetics, to conduct a risk assessment. This assessment could be used for gap filling as well as the prediction of potential mechanisms to guide further investigations. A useful guideline on how AOPs can be integrated into risk assessment was published by Sakuratani and coworkers.^171^ Arzuaga and coworkers showed that the AOP framework can be used to identify and describe possible species‐specific effects.^172^ By introducing information about genetic screens Mortensen et al. could also show that AOPs can be used to gain information about possible susceptibility introduced through genetic variation.^173^ The biggest strength and drawback of AOPs is their linearity. On one hand it introduces simplicity and facilitates usability, on the other hand most AOPs are not limited to a single event chain but can be much more interlinked through common MIEs, modulators or key events. To analyze such dependencies AOP networks were proposed, which can add additional mechanistic information^174^, ^175^ (see Figure 3). Oki and coworkers demonstrated that such networks can be created by mining high throughput screening data in combination with toxicogenomics data, applying frequent itemset mining.^176^

Systems toxicology (following systems biology) is a relatively new discipline, which especially aims to integrate toxicological assessment with novel techniques monitoring changes in the whole organism, such as omics approaches and leverage this information to gain a more complete understanding of toxicological mechanism. Sturla and coworkers aptly describe, “It [Systems Toxicology] will enable the gradual shift from toxicological assessment using solely apical end points toward understanding the biological pathways perturbed by active substances. Systems Toxicology is expected, therefore, to create knowledge regarding both the dynamic interactions between biomolecular components of a complex biological system and how perturbing these interactions with active substances alters homeostasis and leads to adverse reactions and disease.”.^177^ Consequently, systems toxicology will also enable the development of more complex and detailed AOPs and AOP networks that will guide in silico toxicology assessments in the future.

## 6 PITFALLS AND CAVEATS

All methods described in this review have a commonality: all require datasets that can be analyzed or used for model training. Depending on the dataset size some methods might not be feasible. Especially deep learning requires large amounts of data to prevent overfitting. However, if the datasets for the derivation of structural alerts, AOPs or traditional machine learning are small, their applicability also becomes very limited. Read‐across requires the least amount of data with only a few high similarity compounds being sufficient for an extrapolation of the activity. Nevertheless, this specific type of data might be even harder to find, especially for novel structural classes. As all methods build upon data, it is of utmost importance to ascertain that this data contain as few errors as possible, since they have a direct impact on the modeling outcome. Therefore, data curation is essential. Data curation, on one hand, is the removal of uncertain or inconclusive activity values,^178^ and on the other hand, it involves structural curation.^179^, ^180^, ^181^ The removal of data points always is a balancing act between having very few reliable data points or a large dataset with more (less reliable) data points. It always has to be considered carefully and adapted to the method. Whereas deep learning can handle—and might even profit from—noise in the dataset,^182^ for read‐across the activities need to be highly curated and reliable to allow a high certainty in the extrapolation. Structural curation is also necessary to remove duplicates and ensure a similar representation of functional groups.^179^ This, in turn, is needed to ensure a unified calculation of molecular descriptors.

**Figure 4:**
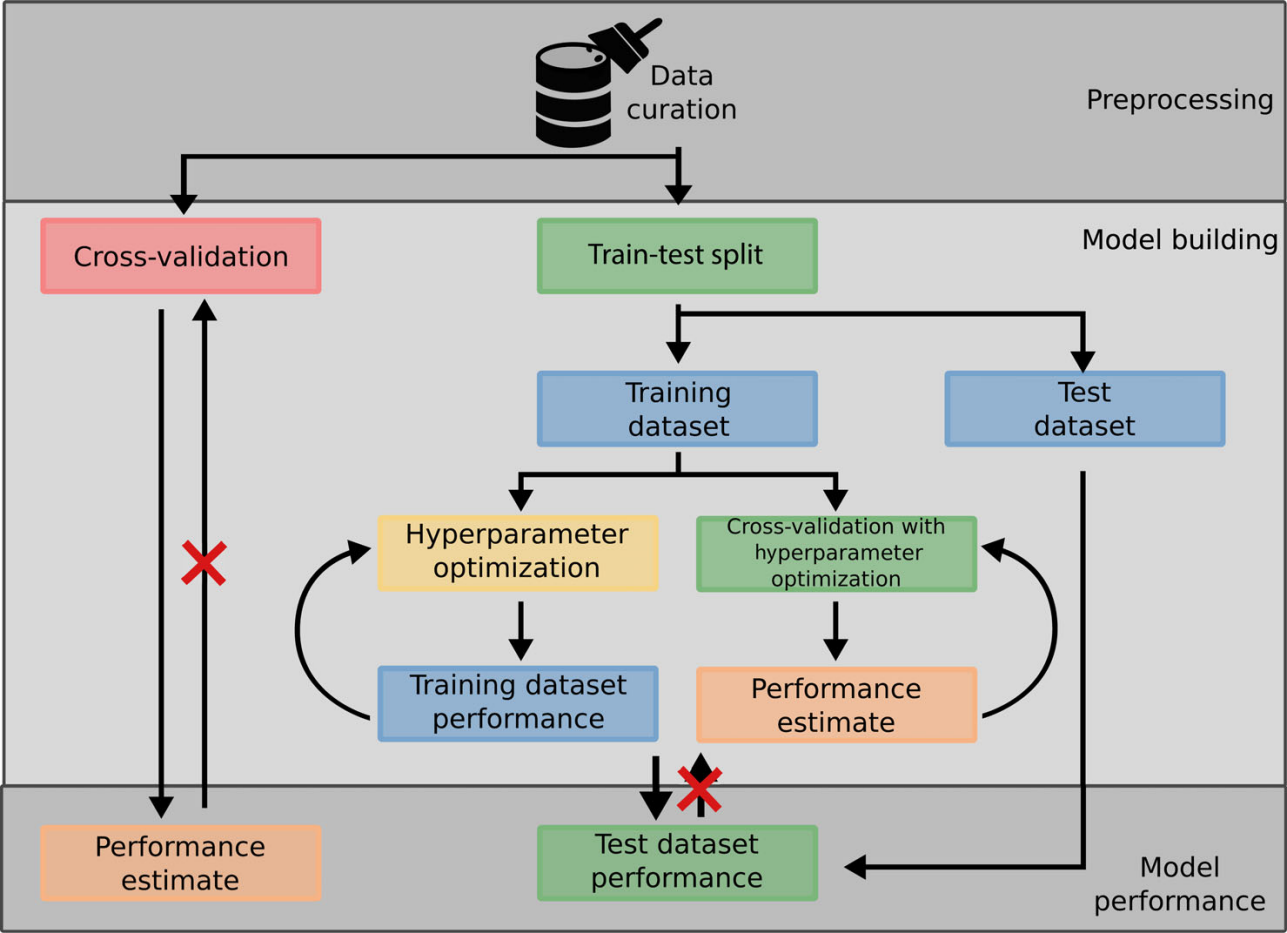
Schematic drawing of possible ways to train a machine learning model. The left path highlights that cross‐validation is possible without splitting the dataset; however, the obtained performance is only an estimate. The left path highlights that a train and test split is needed for example, if hyper‐parameters need to be tuned (in this case second inner cross‐validation can be used) or, in general, if a “real world” situation should be mimicked. It is important to note that, once the test set was used to evaluate the model performance, one should never go back to adjusting parameters with the training set to obtain a better performance on the test set as this would introduce bias into the model

For machine and deep learning, the model evaluation is also crucial. In any case overtraining has to be avoided and the model should be carefully evaluated. Models can be validated by (a) cross‐validation, (b) a train‐test split of the dataset, or (c) an external evaluation dataset (see Figure 4). Cross‐validation is the easiest to use and can be used for cases where data availability is very limited. However, one has to be aware that it only gives an estimate for the model performance and it can be biased by highly similar cross‐validation folds. Therefore, it might not reflect the performance in real‐world scenarios. Train‐test splits are commonly used when the dataset size is large enough to allow for the retention of data solely for testing. With this method, the “real world” performance can be much better estimated, with the drawback that parts of the available data are not used for training. This implies that the model does not benefit from all available information. Just like cross‐validation, it can also be biased by a high similarity between the splits. Thus, the best training method is the evaluation with a true external test set which was compiled independently from the training dataset. Naturally, the external test set has to be within the applicability domain of the model. An interesting approach to first asses the real‐world performance and secondly evaluate the impact of different external datasets is the nested cross‐validation suggested by Baumann and Baumann.^183^ In addition, with the clustered cross‐validation suggested by Mayr et al. in their challenge‐winning DeepTox pipeline,^116^ this could lead to more stable and generalized training of QSAR and deep learning models.

Special care has to be taken when training is conducted with imbalanced datasets. Due to training with greedy algorithms, many machine learning algorithms (including neural networks) tend to ignore the minority class, as the error becomes quite small when all labels are predicted as the majority class. Therefore, the training procedure might have to be modified by the use of cost‐sensitive learning or by over‐ or undersampling techniques.^184^ Examples for such techniques are meta‐cost,^185^ bagging,^186^ or SMOTE.^187^ A model‐independent method to overcome imbalance is conformal predictions as shown by Sun and coworkers.^114^ Lately, it also was shown that for neural networks SMILES enumeration could be a viable method to oversample datasets.^188^ Despite such methods, appropriate evaluation metrics need to be used to determine the models performance. For imbalanced datasets the confusion matrix itself, the Matthews correlation coefficient,^189^, ^190^ the balanced accuracy or the positive or negative predictive value, are appropriate, with the final choice depending on the purpose of the model. The most important measure is always the confusion matrix itself. It already highlights model biases towards a class, which is important especially for imbalanced data. Since from the confusion matrix all other measures can be derived, it just seems logical to always report this for predictive models, along with the modeler's favorite choice of metrics. This allows the reader to in turn calculate their favorite metrics and compare their models to the reported one.

One exception is the area under the receiver operating curve (AUC). This metric cannot be derived from the confusion matrix. However, this already highlights the biggest drawback. It does not evaluate the model at its current state, rather the model's capability regarding different thresholds. However, although the performance at one point might be high, the respective classification threshold is not necessarily useful—just imagine a model with a sensitivity and specificity of 0.8, but a classification threshold of 0.01—here the threshold already highlights the models bias towards one class. Therefore, the AUC should only be used with great care and concomitantly with the confusion matrix to allow an unbiased model evaluation. This brings us back to the OECD validation guidelines for QSAR models. So far, points (1) to (4) were discussed and their importance stressed. The last point—a mechanistic explanation—is probably the hardest to achieve, but might be the most important point to establish confidence in predictions. Whereas structural alerts derived by an expert will mostly have some rational explanation, most automated methods are black‐boxes. For statistically generated structural alerts a literature search can highlight the importance of different structural classes or substructures, while for machine learning methods there is no easy explanation. Although some methods can provide feature importances, no method can yet give a human‐like explanation of a prediction. This is one of the reasons why in silico toxicology is often used as an indicator, but the final call will always be made by expert review, in vitro or in vivo studies. A very comprehensive work, defining how in silico toxicology should be used to become the powerful tool it could be, was published by Myatt and coworkers.^191^

In general, it is important to know how a model was built and especially for which purpose. A model for toxicity prediction in an early stage should not be overly sensitive. In this stage, hazards should be specific to be able to decide if a compound should be advanced. However, a missed alert is not crucial as further tests for safety are conducted. For example, for consumer safety, a missed hazard might lead to subsequent risks. Similarly, the absence of structural alerts never indicates any hazard at all, unless the model was developed to indicate specifically negatives. AOPs are also an example: while they are able to indicate events leading to an apical toxicity endpoint, they are not complete pathways and thus should not be used as such. Especially without any known exposure or kinetic modeling, they only indicate hazards. In fact, different exposures, such as repeated low doses or one‐time high‐dose, could lead to different AOPs. Thus, modifying or compensatory reactions, which are not (yet) part of the AOP framework, can diminish or even abolish the apical toxicity thus negate a possible hazard.

Overall, it can be concluded that all methods have benefits and drawbacks. It is important for modelers and users to specify those and to be aware of limitations, especially during result interpretation.

## 7 WHERE ARE WE HEADED?

A lot has been achieved in the field of in silico toxicology. Emerging technologies are boon and bane. While the modeling gets much more complex with deep learning technologies, it might offer new paths for higher predictivity of models. Especially for ”black‐box” models, which do not allow the users to understand a prediction, the modelers need to build trust. An emerging technology is explainable AI which tries to open the black box. There are many already existing possibilities to explain model prediction, as shown by Polishchuk.^192^ Newer technologies are emerging, especially with regard to neural networks. Two model‐independent approaches are often used: The LIME^193^ and SHAP^194^ frameworks utilize local explainable models to assess the importance of the input feature. In contrast to random forests or regression, this importance is on a per prediction, instead of a per‐model basis. For deep learning, layer wise relevance propagation^195^ is a method that can calculate the contributions of the input features. Many more methods are emerging which could be beneficial for in silico toxicology.^196^ Such methods could generate insights into the structural features or properties which are used by the model. Informing a user about such choices could also improve the trustability. Especially with new techniques such as graph‐based training of neural networks we could gain novel insights into the genesis of a prediction, and maybe leverage such information by discovering novel structural patterns relevant for in silico toxicology.

In addition to better interpretability, studies to evaluate models on a large scale (i.e., across multiple companies, as pursued in the eTOX and eTRANSAFE projects) would not only enhance the confidence, but also improve the models.^110^ However, even with such approaches the current lack of large datasets makes it hard to provide generalized assumptions for modeling or risk assessment. To generate models, as well as read across, or structural alerts with higher generalization potential, data mining and thus a compilation of larger datasets is essential. A drawback is that mined data is never as reliable as manually curated datasets.^197^ However, as stated previously there will always be a trade‐off between reliable data and large datasets. Certainly, with the availability of more data, the amount of reliable data will also increase. An interesting approach could also be the utilization of high throughput screening outcomes as biological fingerprints.^198^, ^199^ Such data is already generated in early drug screening and should, therefore, be leveraged as much as possible.

High throughput data will also enable studies for the combination of structure based and deep learning methods. Lenselink and coworkers showed that proteochemometric modeling, in combination with deep learning, can outperform other approaches for target bioactivity predictions.^200^ Consequently systems toxicology will be able to generalize the modeling approaches not only to singular endpoints but towards an network based prediction. Such approaches could lead to dynamic and computable biological networks, able to predict outcomes based on network perturbances.^177^ The possibilities of such networks are highlighted by Yepiskoposyan and coworkers, who used networks for mucociliary clearance to assess the network perturbance as well as the related mechanism for pyocynanine and IL‐13 treatment.^201^ The relating datasets and information gained from systems toxicology need to be stored in a useful format. In contrast to traditional relational databases, pathway related data is best stored in graph databases which are able to encode the relevant structures such as proteins or genes in the vertices and the relevant relationships such as binding or upregulation, in the edges of the graph.^202^

While we have large public resources for chemical datasets, sadly a plethora of data is only available in proprietary environments. Gedeck and coworkers published an exciting study where models were trained with proprietary data, without the necessity to enclose the structure.^203^ This, in combination with the development of distributed learning,^204^ can also pose a big opportunity to leverage available information in spite of sensitive data.

The AOP framework is also a powerful tool, not only to complement read across or structural alerts, but also to bridge the gaps between all in silico disciplines. If an AOP (or parts of it) is known, respective machine learning models for the MIE(s) or key events could be further used to guide the decision‐making process.

## 8 CONCLUSIONS

In silico toxicology is a cheap and fast tool to detect hazards. Although it is already used within regulatory frameworks, there is still a lot of uncertainty concerning the application and especially the interpretation of such approaches. Once protocols are established and the users get more confident, in silico, along with in vitro tests certainly will in some cases be used as a replacement for in vivo testing. In addition, modelers have to adhere to the OECD guidelines and make sure that the user is informed about the process and intended use of a model. This will establish more trust in models and may overcome the often too rigorous assessment of computational models, as they are expected to outperform the in vitro or in vivo test predictivity. The more we use in silico models the more we can learn about their benefits and limitations and understand where further research could offer new paths.

## CONFLICT OF INTEREST

The authors have declared no conflicts of interest for this article.

## AUTHOR CONTRIBUTIONS

**Jennifer Hemmerich:** Conceptualization; writing‐original draft; writing‐review and editing. **Gerhard Ecker:** Conceptualization; supervision; writing‐original draft; writing‐review and editing.

## RELATED WIREs ARTICLES

Computational toxicology: A tool for all industries

In silico toxicology: Computational methods for the prediction of chemical toxicity

In silico toxicology: Comprehensive benchmarking of multi‐label classification methods applied to chemical toxicity data

## Endnote

1 Here it has to be noted that there were mechanistic toxicology approaches, such as the mode of action concept, which elucidated the mechanisms for specific compounds or compound groups, however, the AOP concept allowed the unification of such approaches into a common framework.
